# Antioxidant Activity and Hepatoprotective Potential of Quercetin 7-Rhamnoside In Vitro and In Vivo

**DOI:** 10.3390/molecules23051188

**Published:** 2018-05-16

**Authors:** Zhi-Qiang Huang, Pan Chen, Wei-Wei Su, Yong-Gang Wang, Hao Wu, Wei Peng, Pei-Bo Li

**Affiliations:** Guangdong Engineering and Technology Research Center for Quality and Efficacy Re-Evaluation of Post-Marketed TCM, Guangdong Key Laboratory of Plant Resources, School of Life Sciences, Sun Yat-sen University, Guangzhou 510275, China; john_ooi@163.com (Z.-Q.H.); chenpan989@126.com (P.C.); lsssww@mail.sysu.edu.cn (W.-W.S.); wangyg@mail.sysu.edu.cn (Y.-G.W.); wuhao_cpu@126.com (H.W.); pweiyu929@126.com (W.P.)

**Keywords:** Quercetin 7-rhamnoside, intracellular antioxidant activity, hepatoprotective, *Hypericum japonicum*

## Abstract

*Hypericum japonicum* is traditionally used as a folk medicine to treat cholestasis and hepatitis. Quercetin 7-rhamnoside (Q7R) is one of the main flavonoid components of *Hypericum japonicum* and has been rarely studied. The aim of the present study was to evaluate the antioxidant activity and hepatoprotective potential of Q7R. In the in vitro experiments, DPPH, ABTS and ferric reducing antioxidant power (FRAP) assays were first performed to assess the antioxidant properties of Q7R, and then a H_2_O_2_-induced oxidative damage cellular model was used to determine the cytoprotective and antioxidant properties of Q7R in human liver L-02 cells. In the in vivo experiment, the hepatoprotective activity of Q7R was evaluated by carbon tetrachloride (CCl_4_)-induced liver damage model in mice. The results of the three in vitro assays (DPPH, ABTS and FRAP) demonstrated that Q7R significantly exhibited antioxidant activity. The cell experiment results showed that Q7R possessed cytoprotective and antioxidant effects on H_2_O_2_-treated L-02 cells. In the in vivo experiments, Q7R suppressed the up-regulation of serum activities of ALT, AST, LDH and triglyceride (TG) levels with dose-dependency. Q7R down-regulated the production of MDA and increased the hepatic GSH content and antioxidant enzymes CAT activities. Hepatic morphological analysis was also performed to confirm the biochemical changes. In summary, these results suggested that Q7R could be considered as a potential source of natural antioxidants, and may become a promising candidate for the treatment of liver injury in the future.

## 1. Introduction

Lots of liver damage, ranging from subclinical icteric hepatitis to necroinflammatory hepatitis, cirrhosis and carcinoma, have been proven to associate with redox imbalance and oxidative stress [1,2]. The liver metabolizes various compounds that produce reactive oxygen radicals (ROS). Oxidative stress is a consequence of discrepancy balance between the production of ROS and antioxidants in a system of defense of human organisms [3]. Recent research in the field of free radical biology suggested an important pathophysiological role of free radicals and oxidative stress in the development and progression of liver diseases [4,5]. Hence, antioxidants are frequently used to treat oxidative liver injury, and the consumption of antioxidants is known to be an important means of preventing or delaying the appearance of liver diseases. Flavonoids, a vast class of hydroxylated phenolic substances, have long been reported as antioxidants in plants, which is due to their ability to scavenge free radicals and to reduce free radical formation [6,7]. Since these compounds are based on the flavan nucleus, the number, positions, and types of substitutions influence radical scavenging and chelating activity [8]. They have multiple biological activities, including antioxidant activity [9], anticarcinogenic, vasodilatory, antiinflammatory, antibacterial, antiallergic, immune-stimulating and antiviral effects [10,11]. The antioxidant activity of flavonoids are due to their capacity to transfer electrons free radicals, chelate metal catalysts, activate antioxidant enzymes, reduce alpha-tocopherol radicals, and inhibit oxidases [12].

**Figure 1:**
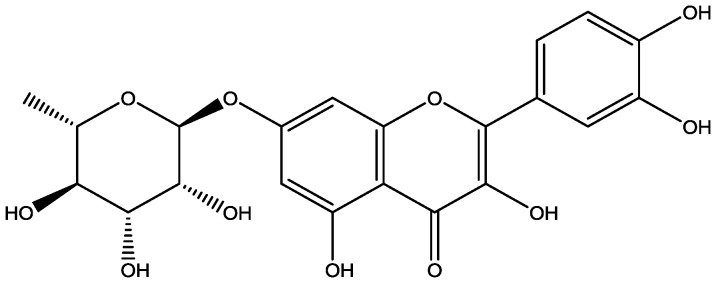
Chemical structure of quercetin 7-rhamnoside (Q7R).

The entire herb of *Hypericum japonicum*, named “Tianjihuang” in China, has been widely used for the treatment of cholestasis, as well as acute and chronic hepatitis [13]. Tianjihuang injection was reported to have significant hepatoprotective effects on carbon tetrachloride (CCl_4_) and acetaminophen-induced acute hepatotoxicity in rats [14]. Amani et al. [15] reported that *Schouwia thebaica* Webb extracts containing Q7R significantly exerted curative effects on CCl_4_-induced liver injury in rats. Q7R (Figure 1) is one of the main flavonoid components of *Hypericum japonicum* [16]. It has been reported that Q7R possesses strong anti-porcine epidemic diarrhea virus activity, which is not simply due to its general action as an antioxidant [17,18]. Our previous study indicated Q7R exhibited hepatoprotective as well as jaundice-relieving effects [19] and could attenuate L-02 cell injury induced by glycochenodeoxycholic acid [13].

In order to figure out the antioxidant capacity of Q7R, a comprehensive investigation based on in vitro methods (DPPH, ABTS, and ferric reducing antioxidant power (FRAP) assays), hepatocyte cellular assay of H_2_O_2_-induced damage and experimental rat model of CCl_4_-induced toxic hepatitis were used in this study.

## 2. Results

### 2.1. Antioxidant Activity by DPPH Method

**Figure 2:**
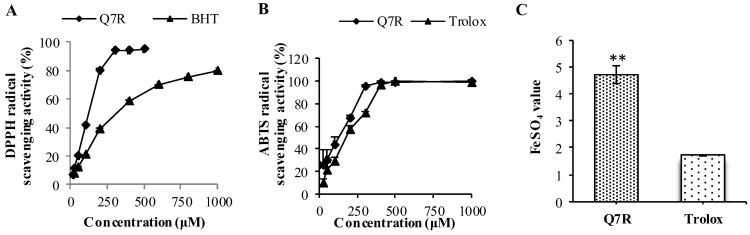
Antioxidant activity of Q7R. The Q7R were examined for DPPH radical scavenging activity (**A**); ABTS radical scavenging activity (**B**); and FRAP (**C**). Values are expressed as mean ± SD of three different experiments performed in triplicate (** *p* < 0.01 were considered significant versus Trolox).

Lower IC_50_ indicates higher antioxidant capacity. In the Figure 2A, Q7R showed stronger activity against H_2_O_2_ (IC_50_ = 118.75 μM) than that of BHT (IC_50_ = 313.69 μM).

### 2.2. Antioxidant Activity by ABTS Method

In ABTS assay, the capacity of Q7R and Trolox to scavenge ABTS is shown in Figure 2B. The EC_50_ value of Q7R is 128.47 μM, much lower than the Trolox with EC_50_ equal to 172.18 μM. It suggests that the antioxidant activity of Q7R was higher than that of Trolox.

### 2.3. Antioxidant Activity by FRAP Method

In FRAP assay, a higher FeSO_4_ value indicates a higher ferric reducing power. The antioxidant activity of Q7R with FeSO_4_ value was shown in Figure 2C, and a higher capacity of reducing ferric capacity was found for Q7R (FeSO_4_ value = 4.72) when compared to Trolox (FeSO_4_ value = 1.75).

### 2.4. Q7R Protected L-02 Cells against H_2_O_2_-Induced Cytoxicity

**Figure 3:**
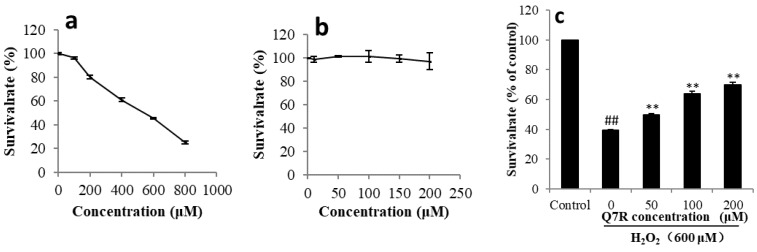
Cytotoxic activity of H_2_O_2_ (**a**) and Q7R (**b**), and Q7R protected against H_2_O_2_-induced cell cytoxicity in L-02 cells (**c**). No H_2_O_2_ and Q7R treatment served as control group. Data were expressed as mean ± standard deviation (SD) of three different experiments performed in triplicate. (## *p* < 0.01 compared to control group. ** *p* < 0.01 compared to cells treated with H_2_O_2_ alone).

As shown in Figure 3, the 50% effective concentration (EC_50_) for 24 h of H_2_O_2_ treatment was 537 μM. Q7R (0–200 μM) showed no cytotoxicity in L-02 cells. As shown in Figure 3c, cell viability is markedly decreased after a 24 h exposure to H_2_O_2_ and pretreatment with Q7R (50, 100 and 200 μM) significantly attenuated H_2_O_2_-induced cytoxity in L-02 cells.

### 2.5. Effect of Q7R on SOD and MDA Level

**Figure 4:**
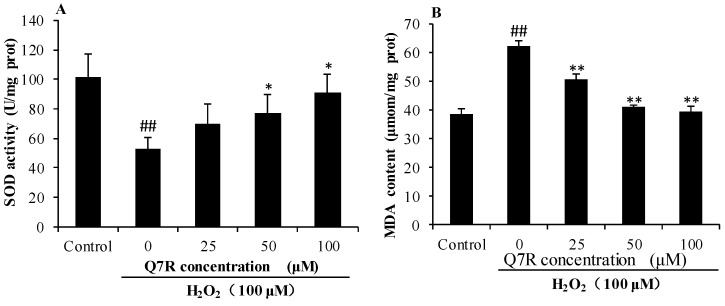
The SOD and MDA levels of normal human liver L-02 cells were served as control group (No H_2_O_2_ and Q7R treatment). The SOD (**A**) and MDA (**B**) levels of L-02 cells were treated with different doses of Q7R before exposure to 100 μM H_2_O_2_ for 24 hours. Bars represent the standard deviation from the mean of three separate experiments. * *p* < 0.05, ** *p* < 0.01, compared to the H_2_O_2_ alone treated group and ## *p* < 0.01, compared to the control group.

As shown in Figure 4, compared with control group, activity of SOD in H_2_O_2_ alone group was significantly decreased (*p* < 0.01). Pretreatment with Q7R (50 and 100 μM) for 1 h could increase activity of SOD. In addition, compared with control group, the level of MDA was significantly increased in H_2_O_2_ alone group (*p* < 0.01), and Q7R (25, 50 and 100 μM) could significantly inhibited the generation of MDA (*p* < 0.01).

### 2.6. Measurement of Serum ALT, AST, LDH and Triglyceride (TG)

**Figure 5:**
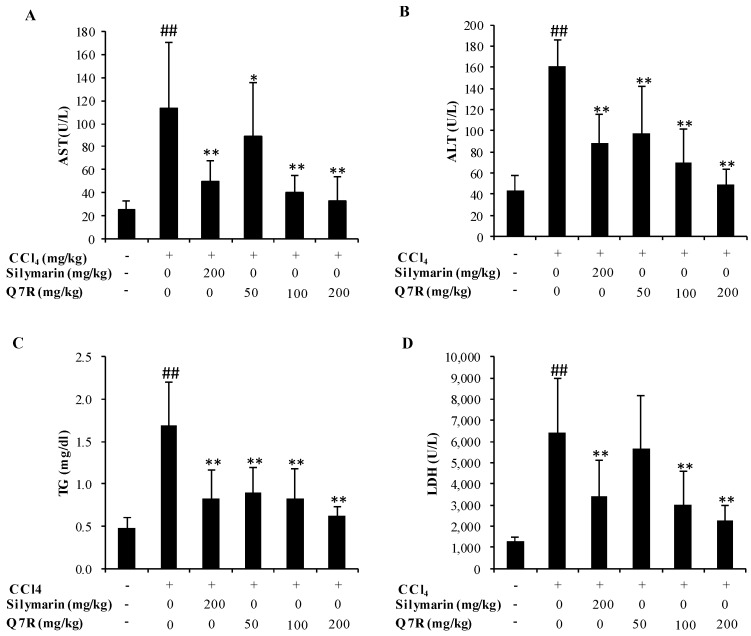
Effects of the Q7R on serum AST (**A**), ALT (**B**), triglyceride (TG) (**C**), LDH (D) in CCl_4_-intoxicated mice. Values express as mean ± SD, *n* = 12. ## *p* < 0.01, compared to normal control group and * *p* < 0.05, ** *p* < 0.01, compared to the CCl_4_ alone treated group.

The effect of Q7R on ALT, AST, LDH and TG are summarized in Figure 5. Mice treated with CCl_4_ alone showed higher serum levels of ALT, AST, LDH and TG as compared to normal control (*p* < 0.05), indicating that CCl_4_-induced hepatotoxicity. Q7R administration caused a significantly reduction of serum levels of ALT, AST, LDH and TG in comparison with those observed in the CCl_4_-treated group (*p* < 0.05). Silymarin (at dose of 200 mg/kg) also reduced the activities of serum enzymes including ALT, AST and LDH and decreased the level of TG. These results suggested that Q7R possessed a potent hepatoprotective action against liver injury induced by CCl_4_.

### 2.7. Measurement of CAT, GSH and MDA in Liver Tissue

**Figure 6:**
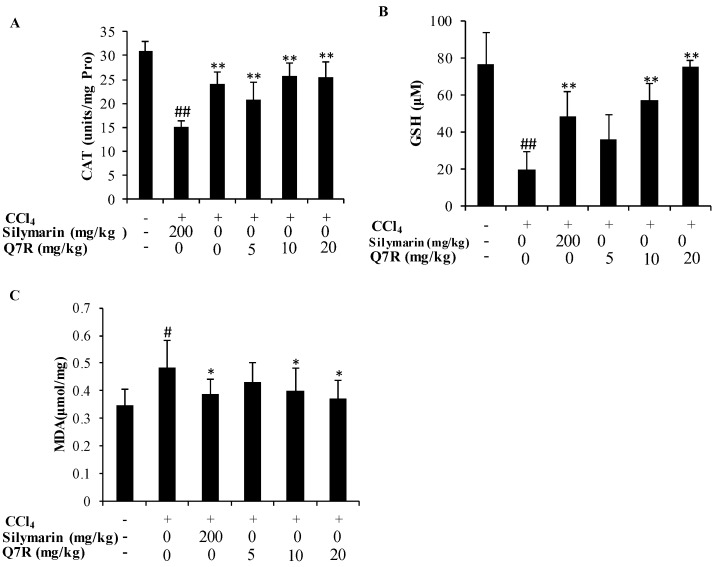
Effect of Q7R on CAT, GSH and MDA levels in liver homogenate. Values expresses as mean ± SD, *n* = 12. # *p* < 0.05, ## *p* < 0.01, compared to normal control group and * *p* < 0.05, ** *p* < 0.01, compared to the CCl_4_ alone treated group.

As shown in Figure 6, the levels of CAT and GSH were conspicuously decreased in CCl_4_ treated mice. Treatment with Q7R and silymarin both reversed such reduction of the activities of CAT and GSH. Animals exposed to CCl_4_ showed significant increase in the levels of MDA (*p* < 0.01). However, the mice were pre-treated with Q7R and silymarin showed significant lower MDA level.

### 2.8. Histopathological Examination

**Figure 7:**
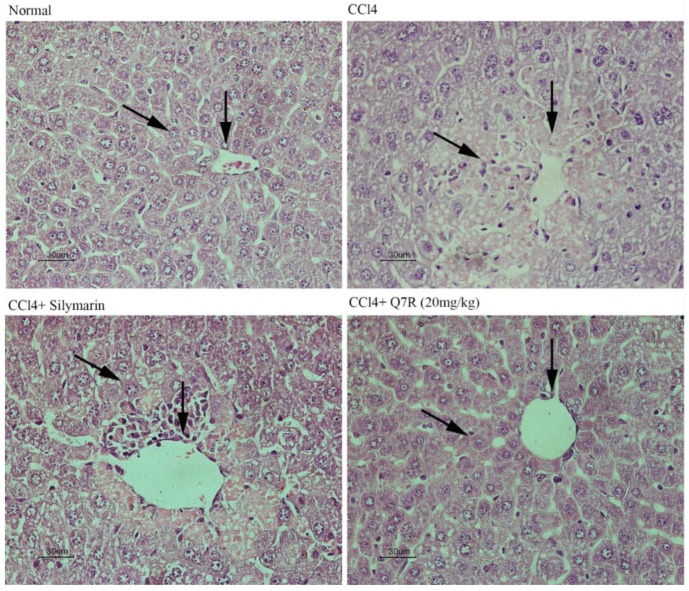
Effects of Q7R on hepatic morphological analysis in CCl_4_-intoxicated mice (Hematoxylin and eosin (H&E)). Note the liver lobular architecture, cell structure and central vein (arrow heads).

Hematoxylin and eosin (H&E) stained sections are shown in Figure 7. Normal liver lobular architecture and cell structure were shown in normal control group. While the liver tissue of the CCl_4_-treated group showed apparent morphological changes including large areas of extensive cell necrosis with loss of hepatocyte architecture around the central vein (CV). Furthermore, some condensed nuclei and massive inflammatory cells infiltration were observed in the injured area. The silymarin (200 mg/kg, i.p.)-treated group showed mild central venous congestion and mild fatty infiltration. The Q7R (20 mg/kg, i.p.)-treated group showed absence of cell necrosis and minimal inflammatory conditions with near-normal liver architecture. Apparently, the Q7R and silymarin pretreatment significantly decreased the injured area, necrotic cells and inflammatory infiltration.

## 3. Discussion

Reactive oxygen species (ROS) are known mediators of intracellular signaling cascades. A balance between oxidant and antioxidant intracellular systems is hence vital for cell function, regulation, and adaptation to diverse growth conditions [20]. Excessive production of ROS may, however, lead to oxidative stress, loss of cell function and ultimately apoptosis or necrosis. This oxidative damage is considered to play a causative role in aging and several degenerative diseases, such as heart disease, cataracts, cognitive dysfunction and cancer [21]. Several reports have indicated that an important mechanism of hepatoprotective effects may be related to their capacity to transfer hydrogen to free radicals, activate antioxidant enzymes and inhibit oxidases [22,23,24,25].

The previous studies indicated that ABTS, DPPH, and FRAP assays as simple and efficient methods could be used to determine antioxidant activity in many plant extracts [26]. Hence, the antioxidant capacities of Q7R were studied using DPPH, ABTS, and FRAP assays first. In fact, BHT and Trolox have been used as a standard antioxidant in the performed experiments [27]. According to the data obtained from the present study, Q7R was found to be an effective antioxidant.

The present study also investigated the potential protective effect and mechanism of Q7R against oxidative stress induced by H_2_O_2_ in human liver L-02 cells. H_2_O_2_, a typical oxidant, has been used to study the response of cells to oxidative stress [28]. MDA is the product of lipid peroxidation and a classic indicator of oxidative stress and SOD is one of the major enzymes responsible for the inactivation of superoxide and hydrogen peroxid [29]. The results of this study showed that Q7R could increase the activity of SOD and inhibit the generation of MDA in L-02 cells.

CCl_4_ has been extensively studied as a liver toxicant. Hepatotoxicity induced by CCl_4_ is the most commonly used model system for screening of hepatoprotective activity of plant extracts and compounds [30]. There is a growing number of researches showed that oxidative stress is an important mechanism for hepatotoxicity induced by CCl_4_ [31,32]. CCl_4_ is biotransformed by the cytochrome P450 system to produce the trichloromethyl free radicals, which in turn covalently bind to cell membranes and organelles to elicit lipid peroxidation [32]. This study evaluated the hepatoprotective effect of Q7R in CCl_4_-induced liver injuries in rat model. Previous work clearly demonstrated that silymarin has anti-inflammatory potential and can alter histopathological changes induced by CCl_4_, such as necrosis, ballooning, and inflammatory infiltration of lymphocytes [33,34,35]. In the present study, silymarin was used as an effective positive control and showed similar results compared with other works [36]. The levels of serum AST, ALT, TG and LDH activities reflects damage to hepatocytes and were considered to be highly sensitive and fairly specific preclinical and clinical biomarkers of hepatotoxicity [37]. In the present study, Q7R could significantly decrease serum levels of AST, ALT, TG, and LDH when compared with the CCl_4_ alone group. The present results are also consistent with the previous reports that Q7R were able to significantly prevent the D-aminogalactose-induced increases in serum enzymes (AST and ALT) and lower ANIT-increased serum total bilirubin levels [19]. To further understand the protective effect of Q7R, this study determined the activities of antioxidant enzymes CAT and GSH, as well as the levels of MDA in mice livers. Since CAT and GSH as antioxidant enzymes were considered to be a primary defense system for oxidative damage prevention [38]. These antioxidant enzymes are effortlessly inactivated by lipid peroxides or free radical, which results in decreased activities of these enzymes in CCl_4_ toxicity. In the present study, treatment with Q7R could enhance the activities of antioxidant enzyme system, including CAT and GSH. The elevation of MDA level in the liver implies enhanced peroxidation leading to tissue damage and breakdown of the antioxidant defense mechanisms [23]. Treatment with Q7R resulted in the reduction of MDA level in CCl_4_-induced liver injury mice, indicating its ability to break the chain reaction of lipid peroxidation. Histological examination of CCl_4_-treated mouse liver showed significant hepatotoxicity characteristics, such as necrosis in hepatic lobules, vacuolization, Kupffer cells around the central vein. However, treatment with Q7R significantly decreased these hepatotoxicity characteristics in mouse liver, suggesting that Q7R provided protection against CCl_4_-induced liver injury. Taking all above together, the results suggested that Q7R possessed a significant hepatoprotective effect on acute CCl_4_-induced liver injury possibly via its antioxidant activity.

In conclusion, the results of this study demonstrate that Q7R isolated from the traditional medicinal plant *Hypericum japonicum* offers hepatoprotection against CCl_4_-induced hepatotoxicity. Our results showed that the hepatoprotective effects of Q7R may be related to its antioxidant activity.

## 4. Materials and Methods

### 4.1. Extraction and Purity of Q7R from Hypericum japonicum

The plant materials were collected from the herbal market, and were authorized as the authentic *Hypericum japonicum* by prof. Wenbo Liao of Sun Yat-sen University. Q7R (98.3% purity) was extracted and purified from *Hypericum japonicum*. Detailed experimental procedures could be seen in our previous work [13]. Chemical structure of Q7R was shown in Figure 1.

### 4.2. Cell Lines and Culture

The immortalized normal liver cell line L-02 was obtained from Animal Experiment Center (Sun Yat-sen University, Guangzhou, China). The L-02 cells were cultured in RPMI-1640 (GIBCO, Carlsbad, CA, USA) medium supplemented with 10% fetal bovine serum (FBS) (Hyclone, Logan, UT, USA), 1% penicillin and 1% streptomycin (GIBCO, USA) at 37 °C in a humidified atmosphere containing 5% CO_2_.

### 4.3. Animals

Kunming mice (22–25 g) of either sex procured from Guangdong Medical Laboratorial Animal Center, PR China. The animals were kept in polypropylene cages and maintained under standard laboratory conditions of a 12-h light/dark cycle and fixed temperature (25 ± 2 °C). They were maintained on water and laboratory chow freely. All experimental procedures were approved by the Animal Care and Use Committee of School of Life Sciences, Sun Yat-sen University, PR China. Adequate measures were taken to minimize pain of experimental animals. 

### 4.4. Chemicals and Reagents

DPPH, Trolox, ABTS, MTT and DMSO of technical grade used in this study were supplied by Sigma Co. (St. Louis, MO, USA). Total Antioxidant Capacity Assay Kit (FRAP method) was supplied by Beyotime Biotechnology Ltd. RPMI-1640 medium, penicillin and streptomycin were purchased from Invitrogen-Gibco (New York, NY, USA). Fetal bovine serum (FBS) was supplied by Hyclone (Hyclone, Logan, UT, USA). SOD, CAT, ALT, AST, T-GSH/GSSG and MDA Kits were supplied by Namjing Jiancheng Bioengineering Institute, Nanjing, China. Silymarin were purchased from Jianmin Pharmaceutical Group Co., Ltd, Wuhan, China.

### 4.5. In Vitro Antioxidant Studies

#### 4.5.1. Antioxidant Activity by DPPH Method

The DPPH assay was performed as described by Clarke et al. [39]. The stock solution was prepared by dissolving 19.7 mg DPPH with 50 mL ethanol and then stored at 4 °C until needed. The Q7R diluted appropriately in ethanol in a concentration range from 12.5 to 500 μM, was mixed with 100 μL of 0.1 mM DPPH in ethanol in wells of 96-well plates. Then, the mixture was incubated at room temperature in a chamber without any light. After incubation for 30 min, the evaluation of the scavenging ability was performed by measuring absorbance at 515 nm in a plate reader (TECAN-Infinite M200). BHT were used as positive control in a concentration range from 25 to 1000 μM. The scavenging activity (%) was calculated using the following equation:

$$\mathrm{DPPH}\;\mathrm{scavenging}\;\left(\%\right)\;=\left[1-(\frac{A_{s}\;-A_{b}}{A_{c}})\right]\times 100$$

where A_c_ is the absorbance of the control (without sample), A_s_ is the absorbance in the presence of the sample, and A_b_ is the absorbance of sample without DPPH radical. The scavenging ability of the samples was expressed as EC_50_ value, which is the effective concentration at which 50% of DPPH radicals were scavenged. The EC_50_ values were calculated from the relationship curve of scavenging activities (%) versus concentrations of respective sample.

#### 4.5.2. Antioxidant Activity by ABTS^∙+^ Method

Radical cation scavenging capacity of Q7R was examined against ABTS^·+^ with some modifications [21]. The preparation of ABTS^∙+^ free radical solution by incubating a mixture of ABTS (7 mM) and K_2_S_2_O_8_ (2.45 mM) was dissolved in distillated water. To create a stable color of radical solution, the mixture was incubated in a dark room at 4 °C for 12 h. Before use, the standard ABTS^∙+^ solution was diluted with ethanol to get the absorption between 0.68 and 0.72 AU at 734 nm. Then, 20 μL of sample at various concentration (25–1000 μM) was mixed with 180 μL of diluted ABTS work solution. Each concentration was analyzed in triplicate after 30 min in a dark condition. Trolox dissolved in pure ethanol was used as positive control. The percentage decrease of absorbance at 734 nm with a microplate reader was calculated for each point. The scavenging activity of the samples was expressed as EC_50_ value, and it was calculated from the relationship curve of scavenging activities (%) versus concentrations of respective sample. The scavenging activity (%) was calculated as:

$${\mathrm{ABTS}}^{\cdot +}\;\mathrm{scavenging}\;(\%)=[1-(\frac{A_{1}\;-\;A_{2}}{A_{0}})]\times 100$$

where A_1_ is the absorbance in the presence of the sample. Sample solution without ABTS working solution was used as a sample blank (A_2_), while ABTS working solution plus ethanol was used as a control blank (A_0_).

#### 4.5.3. Ferric Reducing Assay

Ferric reducing antioxidant power (FRAP) assay was performed according to Luo et al. [40] with some modifications. Q7R was dissolved in ethanol to make a concentration of 1 mM. FRAP kits were used to obtain the FRAP values of the Q7R. FRAP values can be calculated using standard curves (concentration FeSO_4_ of from 0.5 to 5 mM). Trolox was determined as the positive control and the assays were performed as per the manufacturer’s instructions. The analysis was conducted in triplicate for each sample. The results were expressed as FeSO_4_ values, which were calculated as:

$${\mathrm{FeSO}}_{4}\;\mathrm{value}=\frac{\mathrm{FRAP}\;\mathrm{value}}{\mathrm{Concentration}\;\mathrm{of}\;Q7R\;(1\;\mathrm{mM}\;)}$$

#### 4.5.4. Cell Viability Assay

To evaluate the IC_50_ of H_2_O_2_ and the noncytotoxic concentration of Q7R on L-02 cells, the effect of H_2_O_2_ and Q7R on viability of L-02 cell were evaluated using MTT assay respectively. Briefly, the cells were seeded in 96-well culture plate at the concentration of 1 × 10^5^ cells per well in 180 μL of RPMI-1640 medium. After incubation of the cells for 48 h at 37 °C in a humiditied atmosphere of 5% CO_2_, at approximately 80% confluence, various concentrations of H_2_O_2_ and Q7R were respectively added to culture medium for 24 h. Furthermore, the protective effect of Q7R against H_2_O_2_-induced L-02 cell damage also was evaluated using MTT assay. Briefly, cells were pretreated with various noncytotoxic concentration of Q7R for 1 h, and then, cells were treated with H_2_O_2_ at its approximate IC_50_ concentration for 24 h. The cell viability was calculated by the samples of optical densities to the control (medium only) cells. The optical density of the formazan formed in the control cells was taken as 100% viability.

#### 4.5.5. Determination of SOD Activities and MDA Concentration

The SOD activity and MDA concentration were performed according to Kepekci et al [32]. The L-02 cells (1 × 10^5^ cells /mL) were plated in a 96-well plate in RPMI-1640 medium containing 0.1% DMSO and incubated at 37 °C in a humiditied atmosphere of 5% CO_2_. The L-02 cells were treated with various concentration of Q7R for 1 h, and then, H_2_O_2_ (final concentration, 100 μM) were added in the plate for 24 h. The levels of SOD and MDA were determined by commercial assay kits, respectively.

### 4.6. Animal Studies

#### 4.6.1. Treatments

Mice were randomly divided into six experimental groups of 12 animals each. Normal control group and CCl_4_ group received normal saline only (10 mL/kg, i.p.) for 7 days. Silymarin group were pretreated with Silymarin (200 mg/kg/day, in normal saline, p.o.) for 7 days. Q7R groups were pretreated with three different doses of Q7R (5, 10 and 20 mg/kg/day, in normal saline, i.p.) respectively for 7 days. On the 7th day, normal control group received olive oil two hours after the last administration of vehicle. CCl_4_ group, Silymarin group and Q7R groups received 0.2% CCl_4_ (10 ml/kg, in olive oil, i.p.) two hours after the last administration of vehicle, silymarin, or Q7R. Animals were killed 24 h after receiving CCl_4_. Blood samples were collected from the orbital venous plexus. The serum was used for the assay of markers viz., AST, ALT, LDH and TG. Liver samples were dissected out and washed immediately with ice cold saline to remove as much blood as possible, and immediately stored at −80 °C Ultra-low temperature freezer until analysis. An extra sample of liver was excised and fixed in 10% formalin solution for histopathologic analysis.

#### 4.6.2. Measurement of Serum AST, ALT, LDH and TG

The blood was centrifuged at 3000× *g* for 10 min to separate serum. Liver damage was assessed by the estimation of serum activities of ALT and AST, using commercially available test kits provided by Nanjing Jiancheng Bioengineering Institute. In addition, the serum levels of LDH and TG were estimated by Hitachi 7100 automatic biochemical analyzer.

#### 4.6.3. Measurement of CAT, GSH, and MDA in Liver Homogenate

Livers were homogenized and the homogenates were centrifuged at 10,000 r/min for 10 min at 4 °C, and the supernatants were subjected to further measurement. Lipid peroxidation was determined by measuring the thiobar-bituric acid-reactive substances (TBARS) in the homogenate and expressed as MDA concentration. The activities of CAT and GSH were determined according to the instructions supplied with the commercial assay kits.

#### 4.6.4. Histopathological Studies

The livers were preserved in neutral buffered formalin and were processed for paraffin embedding, following the standard microtechnique. Four to five micronsections of livers were stained with haematoxylin and eosin and observed under microscope for the histopathological changes.

#### 4.6.5. Statistical Analysis

Data were expressed as mean ± standard deviation (SD). The significant differences between the groups were assessed by the one-way analysis of variance (ANOVA) using SPSS 20.0, and *p* < 0.05 was considered as significant difference.

## Abbreviations

ROS	Reactive oxygen species
DPPH	1,1-diphenyl-2-picrylhydrazyl
ABTS	2,2′-Azinobis-(3-ethylbenzthiazoline-6-sulphonate)
FRAP	Ferric Reducing Ability of Plasma
BHT	butylated hydroxytoluene
Trolox	6-hydroxy-2,5,7,8-tetramethylchroman-2-carboxylic acid
ALT	Alanine transaminase
AST	glutamic oxalacetic aminopherase
SOD	Superoxide Dismutase
MDA	Malondialdehyde
TG	Triglycerides
LDH	lactate dehydrogenase
GSH	Glutathione
CAT	Catalase
MTT	3-(4,5-Dimethylthiazol-2-yl)-2,5,diphenyl-tetrazolium bromide
DMSO	Dimethyl Sulfoxide
